# Peripheral blood basophils are the main source for early interleukin-4 secretion upon *in vitro* stimulation with *Culicoides* allergen in allergic horses

**DOI:** 10.1371/journal.pone.0252243

**Published:** 2021-05-26

**Authors:** Fahad Raza, Susanna Babasyan, Elisabeth M. Larson, Heather S. Freer, Christiane L. Schnabel, Bettina Wagner

**Affiliations:** Departments of Population Medicine and Diagnostic Sciences, College of Veterinary Medicine, Cornell University, Ithaca, New York, United States of America; Midwestern University, UNITED STATES

## Abstract

Interleukin-4 (IL-4) is a key cytokine secreted by type 2 T helper (Th2) cells that orchestrates immune responses during allergic reactions. Human and mouse studies additionally suggest that basophils have a unique role in the regulation of allergic diseases by providing initial IL-4 to drive T cell development towards the Th2 phenotype. Equine *Culicoides* hypersensitivity (CH) is a seasonal immunoglobulin E (IgE)-mediated allergic dermatitis in horses in response to salivary allergens from *Culicoides* (*Cul*) midges. Here, we analyzed IL-4 production in peripheral blood mononuclear cells (PBMC) of CH affected (n = 8) and healthy horses (n = 8) living together in an environment with natural *Cul* exposure. During *Cul* exposure when allergic horses had clinical allergy, IL-4 secretion from PBMC after stimulation with *Cul* extract was similar between healthy and CH affected horses. In contrast, allergic horses had higher IL-4 secretion from PBMC than healthy horses during months without allergen exposure. In addition, allergic horses had increased percentages of IL-4^+^ cells after *Cul* stimulation compared to healthy horses, while both groups had similar percentages of IL-4^+^ cells following IgE crosslinking. The IL-4^+^ cells were subsequently characterized using different cell surface markers as basophils, while very few allergen-specific CD4^+^ cells were detected in PBMC after *Cul* extract stimulation. Similarly, IgE crosslinking by anti-IgE triggered basophils to produce IL-4 in all horses. PMA/ionomycin consistently induced high percentages of IL-4^+^ Th2 cells in both groups confirming that T cells of all horses studied were capable of IL-4 production. In conclusion, peripheral blood basophils produced high amounts of IL-4 in allergic horses after stimulation with *Cul* allergens, and allergic horses also maintained higher basophil percentages throughout the year than healthy horses. These new findings suggest that peripheral blood basophils may play a yet underestimated role in innate IL-4 production upon allergen activation in horses with CH. Basophil-derived IL-4 might be a crucial early signal for immune induction, modulating of immune responses towards Th2 immunity and IgE production.

## Introduction

Immunoglobulin E (IgE)-mediated allergies, such as hay fever, atopic dermatitis, or food allergies, affect people worldwide [1–5] and also animals such as dogs, cats and horses. Allergy symptoms can range from discomfort to life-threatening anaphylaxis [3–6]. Binding of IgE to high-affinity IgE receptors (FcεR1) on the surface of mast cells, a process called sensitization, precedes clinical allergy and is crucial for its development [7, 8]. During an allergic reaction, allergens crosslink IgE molecules on the surface of mast cells. This triggers the immediate release of histamine and other inflammatory mediators from these cells and initiates innate and adaptive immune mechanisms [7, 9, 10].

Horses develop a seasonal recurrent allergic dermatitis, called *Culicoides* hypersensitivity (CH). The disease is also known as summer eczema, sweet itch, summer seasonal recurrent dermatitis, insect bite hypersensitivity and Queensland itch [8, 11]. CH is the most common allergic disease of horses and occurs worldwide with the exception of Iceland, New Zealand and Antarctica. CH affects adult horses of all breeds [12–18], including Thoroughbreds, Arabian horses, Warmbloods, Draft horses, Quarter horses, Friesian horses, different pony breeds and many others [11]. Wide variations in prevalence of CH were observed in different countries and can be explained by multiple factors influencing the disease such as the horse’s environment, genetic predisposition, and age [11, 13]. Major modifiers of disease prevalence are the risk of exposure to *Culicoides (Cul)* midges and the age of the horse when it is first exposed to *Cul*. The influence of the initial exposure time to *Cul* became obvious in Icelandic horses. In Iceland, *Cul* midges do not occur, the allergy does not exist, and clinical signs exclusively develop after importation of horses to *Cul* endemic countries, e.g. Europe or the US [11, 13]. Epidemiological studies confirmed that between 26–72% of the exported Icelandic horses developed the disease, while only 7–27% of Icelandic horses born in Europe became affected with CH [11, 13, 15, 16].

CH is an IgE-mediated allergy [7] in response to salivary proteins of biting *Culicoides (Cul)* midges [7, 13, 19]. *Cul* are typically present in the environment during the summer [11, 20, 21], and during *Cul* exposure, allergic horses develop pruritus, alopecia, dermatitis, allergic wounds and, in severe cases, weight loss while their healthy herd mates gain weight [20, 22–24]. Clinical signs start to resolve as soon as *Cul* midges are not present in environment anymore and allergic horse often appear clinically healthy during the winter [10, 11]. Current therapeutics for CH are at best providing some symptomatic relief for affected horses. There is currently no treatment that cures CH.

The causal involvement of IgE in CH was first demonstrated by a modified Prausnitz-Kustner experiment: IgE from allergic horses was transferred into the skin of healthy recipients and after intradermal (i.d.) *Cul* injections, an immediate skin reaction developed in the healthy horses [7]. The immediate skin reaction required allergen-specific IgE crosslinking on the surface of sensitized mast cells and was not observed after i.d. *Cul* injection alone in healthy horses [7]. In general, crosslinking of allergen specific IgE/FcεRI on mast cells results in degranulation, release of histamine, other inflammatory mediators, and cytokines like IL-4, IL-9, IL-13 and TNF-α [25].

Interleukin-4 (IL-4) is a major cytokine secreted by T-helper type 2 (Th2) and supports immunoglobulin class switching to IgE, and thereby development of allergy [26–28]. IL-4 is also considered critical during the development of an allergen-specific Th2 response [29–32] and plays important immunoregulatory functions on immune cells (B-cells, T-cells, monocytes and dendritic cells) and non-immune cells (endothelial cells and fibroblasts) [33–35]. The initial IL-4 signal that leads to the differentiation of naïve T-cells into Th2 cells is debated to either come from T-cells themselves, or from basophils or NKT-cells [36]. However, once Th2 cells developed during an allergic reaction, additional allergen exposure further promotes the production of IL-4 and provides a positive feed-back loop to maintain and/or expand allergen-specific Th2 cells [32, 37, 38].

In addition, innate immune cells such as macrophages, monocytes, eosinophils, mast cells and basophils can produce IL-4 in humans [39–44], mice [41–47] and horses [48]. Basophils are rare myeloid cells in peripheral blood, totaling less than 1% of the white blood cells [46, 48, 49]. Similar to mast cells, basophils express high-affinity FcεR1 on their surface which binds IgE [7–10, 13, 16, 31, 33, 48] and crosslinking of the receptor bound IgE leads to subsequent inflammatory mediator release [7–10, 31, 33]. In horses, Th2 cells produce IL-4 in response to PMA/ionomycin stimulation [50]. In contrast, anti-IgE stimulation of PBMC in both adult horses and neonates results in IL-4 production from IgE^+^ cells [48]. Further characterization of the IL-4^+^/IgE^+^ cells showed that these were IgE^+^/MHCII^low^/CD14^-^ peripheral blood basophils [48].

In this article, we analyzed IL-4 production by peripheral blood cells in horses with CH and healthy control horses monthly for one year to identify whether IL-4 production was different between the two groups with and/or without environmental allergen exposure, and to characterize IL-4^+^ cells in PBMC of horses.

## Materials and methods

### Horses, *Culicoides* exposure and clinical allergy scoring

This study included sixteen adult Icelandic horses that were either allergic with CH (n = 8, 7 mares and 1 gelding) or non-allergic (n = 8, all mares). The majority of these horses were imported in 2012, 2013, or 2016 from Iceland to the United States (Cornell University, Ithaca, New York), and some were born and raised at Cornell University. Allergic horses were further divided into two groups; 1) Allergic for more than five years (>5Y, n = 4) with a median age of 14 years (range 13–15 years) and 2) allergic for less than five years (<5Y, n = 4) with a median age of 8 years (range 7–13 years). The non-allergic horses had a median age of 6.5 years (range 5–12 years) (Table 1).

**Table 1:** Clinical allergy scores ^a^ of study groups during natural exposure to *Culicoides (Cul)* midges in the environment (mid-May to mid-October)^b^.

	Horses	Sex	Year of birth	Age ^c^ (Years)	Allergy score ^d^ during *Cul* exposure						Median (range)
					MAY	JUN	JUL	AUG	SEP	OCT	
Allergic	1	Mare	2005	13	0.3	3.5	3.5	3.7	3.0	1.0	3.5 (0.3–3.7)
	2	Mare	2004	14	2.0	6.0	7.0	7.0	6.0	3.0	6.5 (2–7)
	3	Mare	2004	14	2.3	5.0	6.5	6.7	5.0	2.0	5.8 (2–6.7)
	4	Mare	2003	15	2.0	7.0	8.0	6.7	6.0	1.0	6.8 (1–8)
	5	Mare	2005	13	1.7	5.5	7.5	5.0	5.0	2.0	5.3 (1.7–7.5)
	6	Mare	2009	9	1.3	3.5	5.5	6.0	5.0	2.0	5.3 (1.3–6)
	7	Mare	2011	7	0.0	3.0	3.0	3.0	0.0	0.0	3.0 (0–3)
	8	Gelding	2011	7	1.0	3.5	5.0	6.3	5.0	2.0	5.0 (1–6.3)
Non-allergic	9	Mare	2007	11	0	3	0.5	0	0	0	0.3 (0–3)
	10	Mare	2006	12	0.0	1.5	1.0	0.0	0.0	0.0	0.5 (0–1.5)
	11	Mare	2013	5	0.3	2.0	1.0	0.3	0.0	0.0	0.7 (0–2)
	12	Mare	2011	7	0.3	0.5	0.0	1.3	2.0	0.0	0.9 (0–2)
	13	Mare	2011	7	0.0	0.5	0.0	0.0	0.0	0.0	0.0 (0–0.5)
	14	Mare	2012	6	0.3	0.5	1.0	0.7	0.0	0.0	0.6 (0–1)
	15	Mare	2013	5	0.0	0.5	0.0	0.0	0.0	0.0	0.0 (0–0.5)
	16	Mare	2013	5	1.0	3.0	2.0	2.0	0.0	0.0	2.0 (0–3)

^a^ Clinical allergy scores ranged from 0–10 as described by Miller et al. 2019 [51].

^b^ From January to mid-May and mid-October to December *Cul* midges were not present in the environment where horses were kept.

^c^ Age in years at time of study, in 2018.

^d^ Average allergy score at time of study, in 2018.

All horses were kept in the same environment at Cornell University. They were housed outside 24/7 with free access to run-in-sheds, water and salt blocks. Horses were on large pasture during the summer and were fed grass hay in late fall and winter without any other supplementary feeding. All horses were naturally exposed to environmental *Cul* midges from mid-May to mid-October. Allergic horses showed clinical signs of CH during the summer of the study period and in previous summers, resulting in clinical allergy scores of ≥ 3 as previously described [51, 52]. Clinical allergy scoring was performed 2 to 4 times a month. Throughout the study period, a single individual experienced in allergy scoring assigned the scores using the scoring system shown in S1 Table.

Average monthly scores and median scores and ranges were calculated for individual horses for the months of natural exposure to *Cul* in the environment (May to October) (Table 1) and between January to April, November and December when *Cul* did not occur in the environment of the horses (S2 Table). All horses were dewormed with moxidectin/praziquantel in December of the year prior to this study and then again after the last samples were taken in December (2018). They were not dewormed while the study was ongoing. All horses were vaccinated against rabies, tetanus, Eastern and Western Encephalitis virus and West Nile virus but not vaccinated or treated otherwise during the study interval.

### Blood sample collection

Monthly blood samples were taken from the jugular vein using heparinized vacutainer tubes (Becton-Dickinson, Franklin Lakes, NJ) between April to December. All samples were collected early morning and processed on the same day. All experimental procedures involving animals were in accordance with the National Institute of Health’s guidelines and were approved by the Cornell University Institutional Animal Care and Use Committee (protocol #2011–0011). The study also followed the Guide for Care and Use of Animals in Agriculture Research and Teaching.

### PBMC isolation, stimulation, and measurement of IL-4 secretion

Density gradient centrifugation (Ficoll-Paque^™^ Plus, GE Healthcare, Piscataway, NJ) was used to isolate PBMC from heparinized blood as previously described [48, 50]. A total of 6 x 10^5^ cells/well were cultured in flat bottom 96 well plates (Corning Incorporated, Kennebunk, ME) using cell culture medium (DMEM with, 1% non-essential amino acids, 2mM L-glutamine, 50 μM 2-mercaptoethanol, 50 μg/ml gentamycin, 100 U/ml penicillin, 100μg/ml streptomycin (Thermo Fisher Scientific, Waltham, MA) and 10% fetal calf serum (Atlanta Biological, Flowery Branch, GA). PBMC were kept in medium without stimulation or were stimulated with either 1 μg/ml anti-equine IgE-134 [53], or a 1:5 dilution of *Cul* extract (Stallergenes Greer Inc., Cambridge, MA) or 4 μg/ml phytohemagglutinin (PHA) (Sigma, St. Louis, MO) in cell culture medium. Prior to using it for PBMC stimulation, *Cul* extracts was dialyzed overnight with normal saline to remove organic solvents and bioactive amines. Cell supernatants from PBMC were harvested after 24 and 48 hours of incubation at 37°C in 5% CO_2_. A total of 50 μl of each supernatant was used to detect IL-4 secretion from PBMC by a bead-based assay as previously described [54]. Supernatants from PBMC stimulated with PHA were used as a cell viability control as indicated by high IL-4 secretion from all PHA stimulated samples. All IL-4 concentrations from samples stimulated by anti-IgE and *Cul* extract were corrected by the spontaneously released IL-4 concentrations from PBMC cultured in medium alone from the respective horse and PBMC sampling date.

### Basophil quantification in PBMC

Equine peripheral blood basophils have previously been described as IgE^+^/MHCII^low^/CD14^-^ cells [48, 50]. A total of 6 x 10^6^ PBMC per horse were fixed in 2% (v/v) formaldehyde (Sigma Aldrich), washed twice in PBS and stored in PBS/BSA (phosphate buffer saline, 0.5% (w/v) bovine serum albumin and 0.02% (w/v) sodium azide (VWR International, Radnor, PA) at 4°C in the dark until staining within 24 hours. For cell surface staining, 1 x 10^6^ fixed PBMC were incubated in 50 μl of antibody solution in PBS/BSA. The staining was performed in two steps. First, a mixture of monoclonal antibodies (mAbs) to equine IgE clone 176 [53] and equine MHC class II clone cz11 [55] were incubated with the cells for 15 minutes. The IgE mAb was conjugated with Alexa fluorochrome 488 (A488) and the MHCII mAb was biotinylated. After incubation, cells were washed once with PBS/BSA and then incubated with Streptavidin-Phycoerythrin (PE) for 10 minutes. Cells were washed with PBS/BSA one more time. All samples were measured using a FACS Canto II flow cytometer (Becton-Dickinson Biosciences, San Diego, CA) counting 50,000 events per sample.

### Intracellular staining and phenotyping of IL-4^+^ cells

To phenotype IL-4^+^ cells, PBMC were isolated from all 16 horses in August. Isolated PBMC (6 x 10^6^ cells) were stimulated with 1 μg/ml anti-equine IgE clone 134 [53] or with a 1:5 dilution of *Cul* extract, prepared as described above, in 24 well plates. Cell culture medium or stimulation with 25 ng/ml of phorbol 12-myristate 13-acetate (PMA)/1μM of ionomycin (Sigma, St. Louis, MO) served as negative or positive controls respectively. PBMC were incubated for 4 hours in the presence of 10 μg/ml of Brefeldin A (Sigma, St. Louis, MO) to block protein secretion. After stimulation, cells were collected, washed once with PBS and fixed as described above.

Initially, time kinetics were performed for allergen-specific stimulation of the PBMC with *Cul* extract to identify the preferred incubation time for flow cytometric analysis of IL-4^+^ innate immune cells. First, PBMC from two allergic horses were incubated for 2, 4, 8, 16, 20, 24, 28, 32 and 48 hours with the protein secretion blocker Brefeldin A added for the entire incubation time. Second, PBMC from allergic (n = 5) and non-allergic horses (n = 5) were incubated for 4, 8, 16, 24, 32 and 48 hours with Brefeldin A added only for the last four hours of incubation.

Intracellular IL-4 staining was performed by incubation with anti-equine IL-4 mAb clone 13G7 [56] in saponin buffer (0.5% (w/v) saponin (Sigma, St. Louis, MO) in PBS/BSA), or an isotype control (unpublished mAb). Both the IL-4 mAb and isotype control were conjugated to A647. Along with the IL-4 mAb or isotype control, combinations of two additional mAbs were included to phenotype IL-4^+^ cells. These mAb combinations were (i) equine IgE (clone 176) and equine MHCII to identify basophils (IgE^+^/ MHCII^low^); (ii) equine CD4 clone HB61A [57] and equine CD8 clone CVS8 [56] to identify T-cells (CD4^+^ and/or CD8^+^); (iii) equine IgM clone 1–22 [58] and equine IgG1 clone CVS45 [59] to identify B-cells (IgM^+^ and/or IgG1^+^); and (iv) equine CD14 clone 105 [60] and equine IgE (clone 176) to identify monocytes (CD14^+^) and IgE^+^ monocytes (IgE^+^/CD14^low^). For all combinations, one mAb was conjugated to A488 and the other mAb was biotinylated (Table 2). Samples were simultaneously stained with all three mAbs in saponin buffer for 15 minutes. The cells were then washed twice with saponin buffer and once with PBS/BSA and afterwards incubated with streptavidin-phycoerythrin in PBS/BSA for 10 minutes. After a final wash in PBS/BSA, the samples were measured in a FACS Canto II flow cytometer (Becton-Dickinson Biosciences, San Diego, CA) by detecting 100,000 events.

**Table 2:** Equine monoclonal antibodies against cell surface antigens and intracellular IL-4 used for staining of PBMC and flow cytometric analysis.

	Cell population	Antibody (clone)	Antigen	Fluorochrome/ Biotin	Reference
i	Basophils	IgE (176)	Cell surface	A488	[53]
		MHCII (cz.11)	Cell surface	Biotin	[55]
		IL-4 (13G7)	Intracellular	A647	[56]
ii	T-cells	CD4 (HB61A)	Cell surface	A647	[57]
		CD8 (CVS8)	Cell surface	Biotin	[57]
		IL-4 (13G7)	Intracellular	A488	[56]
iii	B-cells	IgM (1–22)	Cell surface	A488	[58]
		IgG1 (CVS45)	Cell surface	Biotin	[59]
		IL-4 (13G7)	Intracellular	A647	[56]
iv	Monocytes	CD14 (105)	Cell surface	Biotin	[60]
		IgE (176)	Cell surface	A488	[53]
		IL-4 (13G7)	Intracellular	A647	[56]

### Statistical analysis

Data were tested for normality and were not normally distributed; therefore, a non-parametric Mann-Whitney test was performed with a 95% confidence interval to compare IL-4 secretion and basophil percentage between allergic and non-allergic horse groups. For phenotyping of IL-4^+^ cells, Wilcoxon matched-pair signed rank tests with a 95% confidence interval were used to compare IL-4^+^ cells in response to different stimuli. A p-value of <0.05 was considered significant. All statistical analyses were performed using GraphPad Prism, Version 6 (GraphPad Software, La Jolla, CA).

## Results

### Stimulation of PBMC with anti-IgE or *Culicoides* induces IL-4 secretion

**Fig 1:**
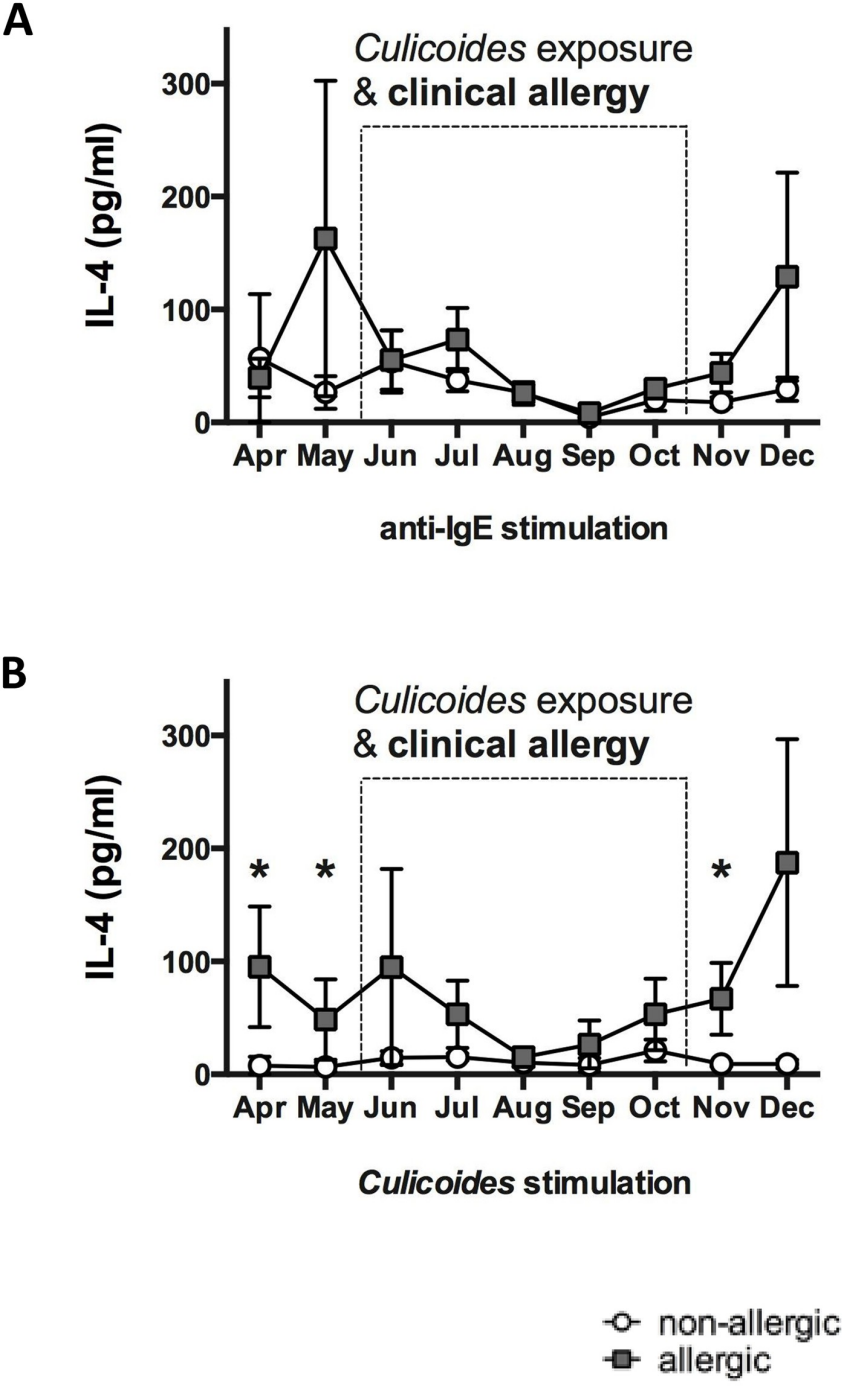
Anti-IgE and *Culicoides (Cul)* stimulation induced IL-4 secretion in PBMC of allergic and non-allergic horses. Blood samples were obtained monthly from allergic (n = 8) and non-allergic horses (n = 8) from April to December. The dotted box indicates the period of environmental exposure to *Cul* when horses with CH showed clinical signs of allergy. PBMC were stimulated *in vitro* with different stimuli, cell culture supernatants were harvested after 24 hours of incubation, and IL-4 was measured in the supernatants using a fluorescent bead-based IL-4 assay. IL-4 secretion from PBMC after stimulation with A) anti-IgE, clone 134, which crosslinks IgE on the cell surface, and B) *Cul* extract. Graphs represent means with standard errors. Allergic and non-allergic groups were compared using non-parametric Mann Whitney tests. * p<0.05.

Horses with CH and non-allergic control horses living in the same environment with natural exposure to *Cul* midges were analyzed for differences in their peripheral blood cell IL-4 secretion. Monthly samples were collected for nine months while clinical signs of allergy were either present or absent in the CH group. PBMC were isolated and stimulated *in vitro* with anti-IgE or *Cul* and IL-4 secretion was measured in the cell culture supernatants. Stimulation of PBMC with the crosslinking IgE mAb 134 induced secretion of IL-4 in similar concentrations in allergic and non-allergic horses (Fig 1A). This suggested that PBMC from allergic and non-allergic horses were equally capable of secreting IL-4 in response to IgE crosslinking.

To determine if horses with CH had increased allergen-specific IL-4 responses, we stimulated PBMC with *Cul* (Fig 1B). A general trend of higher IL-4 secretion was observed from PBMC of allergic horses after 24 hours of stimulation. IL-4 secretion after *in vitro Cul* stimulation was also significantly increased in allergic horses while *Cul* midges were absent from the environment, i.e. in April (*p* = 0.0221), May (*p* = 0.0241) and November (*p* = 0.0103). During *Cul* exposure and while allergic horses showed clinical allergy, differences in IL-4 production were not observed between the two groups. After 48 hours of stimulation, the concentration of secreted IL-4 in PBMC supernatants increased only slightly (S3 Table) but were similar between the allergic and non-allergic groups (S1 Fig). This suggested that the initially secreted IL-4 may have originated from innate immune cells.

**Fig 2:**
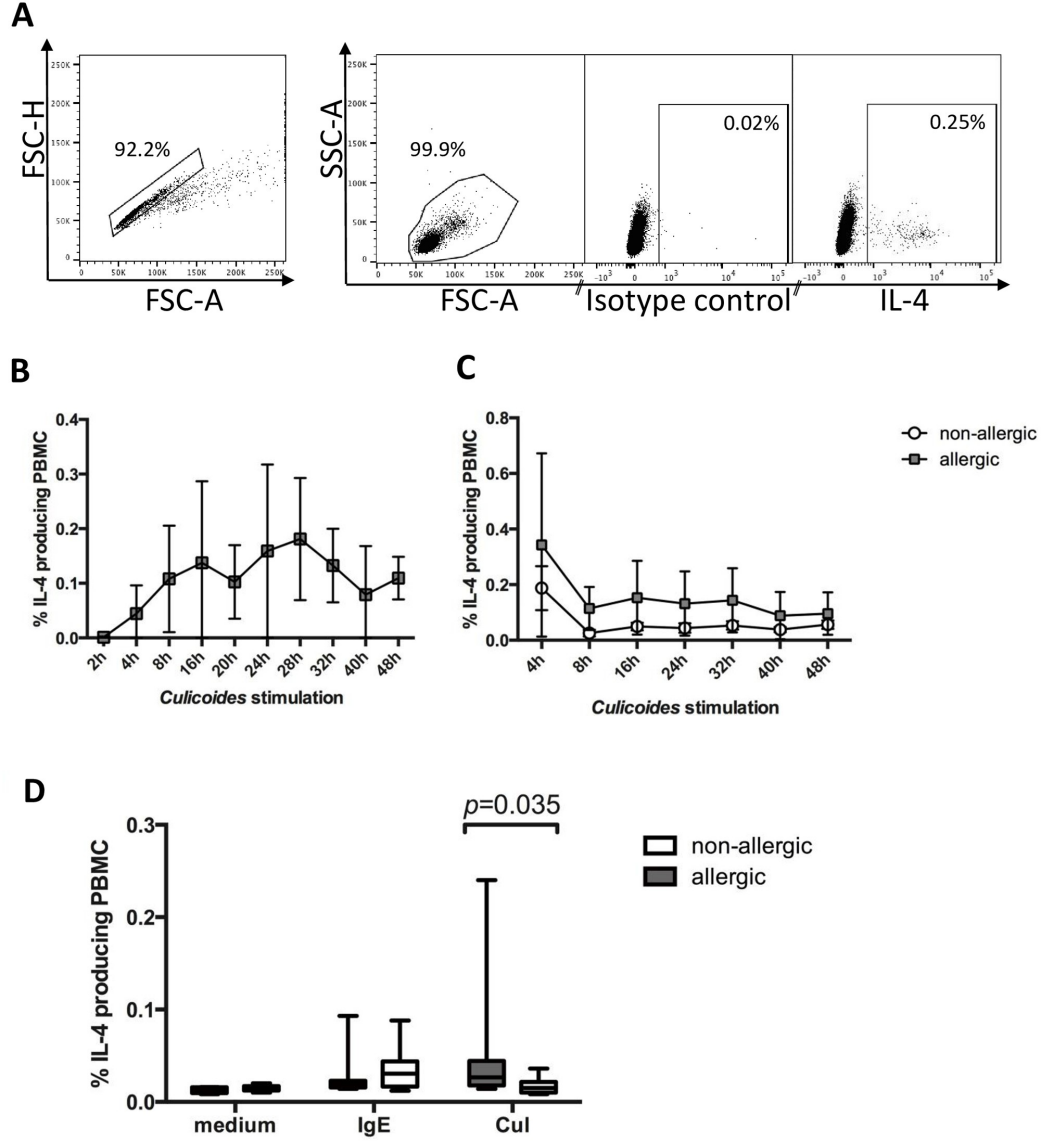
Flow cytometric analysis of IL-4^+^ cells after stimulation of PBMC from allergic and non-allergic horses with anti-IgE or *Cul* extract. PBMC were stimulated *in vitro* with crosslinking anti-IgE mAb 134 or *Cul* in the presence of the secretion blocker Brefeldin A. After incubation, the cells were fixed, stained for intracellular IL-4 production, and analyzed by flow cytometry. A) Gates were set for doublet exclusion (FSC-H/FSC-A) and on PMBC by forward and side scatter characteristics (FSC-A/SSC-A). PBMC were then analyzed for IL-4 expression in comparison to the isotype control. B) PBMC from two allergic horses were stimulated with *Cul* in the presence of the secretion blocker Brefeldin A for different times between 2–48 hours. Cells were fixed after incubation and stained for intracellular IL-4. C) PBMC from allergic (n = 5) and non-allergic horses (n = 5) were stimulated for 4–48 hours with *Cul* and Brefeldin A was only added for the last four hours of incubation. D) Percentages of IL-4^+^ PBMC from horses with CH (n = 8) and non-allergic controls (n = 8) were isolated in August, stimulated for four hours with anti-IgE 134 or *Cul* or kept in medium as control.

Our previous and present studies have shown that allergic horses in upstate NY have the highest clinical allergy scores in July and August [51, 52]. Due to re-activation of allergen-specific IgE^+^ B- cells after re-exposure to *Cul* allergen in early summer, we expected high amounts of *Cul*-specific IgE on the cell surface of innate immune cells during the peak of clinical allergy. PBMC were thus obtained in August to compared percentages of IL-4^+^ cells in PBMC from allergic and non-allergic horses after stimulation with anti-IgE or *Cul* by flow cytometric analysis (Fig 2). Time kinetics of PBMC after *Cul* stimulation indicated that IL-4^+^ cells can be detected between 4–48 hours of stimulation with the secretion blocker Brefeldin A present for the whole incubation time (Fig 2B). However, if Brefeldin A was only present in the stimulation cultures for the last four hours, the highest IL-4^+^ cells percentages were found after four hours of incubation (Fig 2C). The latter incubation time was thus selected to further investigate early IL-4 production in equine PBMC. In agreement with the IL-4 secretion results above, anti-IgE induced similar percentages of IL-4^+^ cells in allergic and non-allergic horses, while IL-4^+^ cells were increased in the allergic group after *Cul* stimulation (p = 0.0350; Fig 2D).

### Basophils in peripheral blood of allergic and non-allergic horses

**Fig 3:**
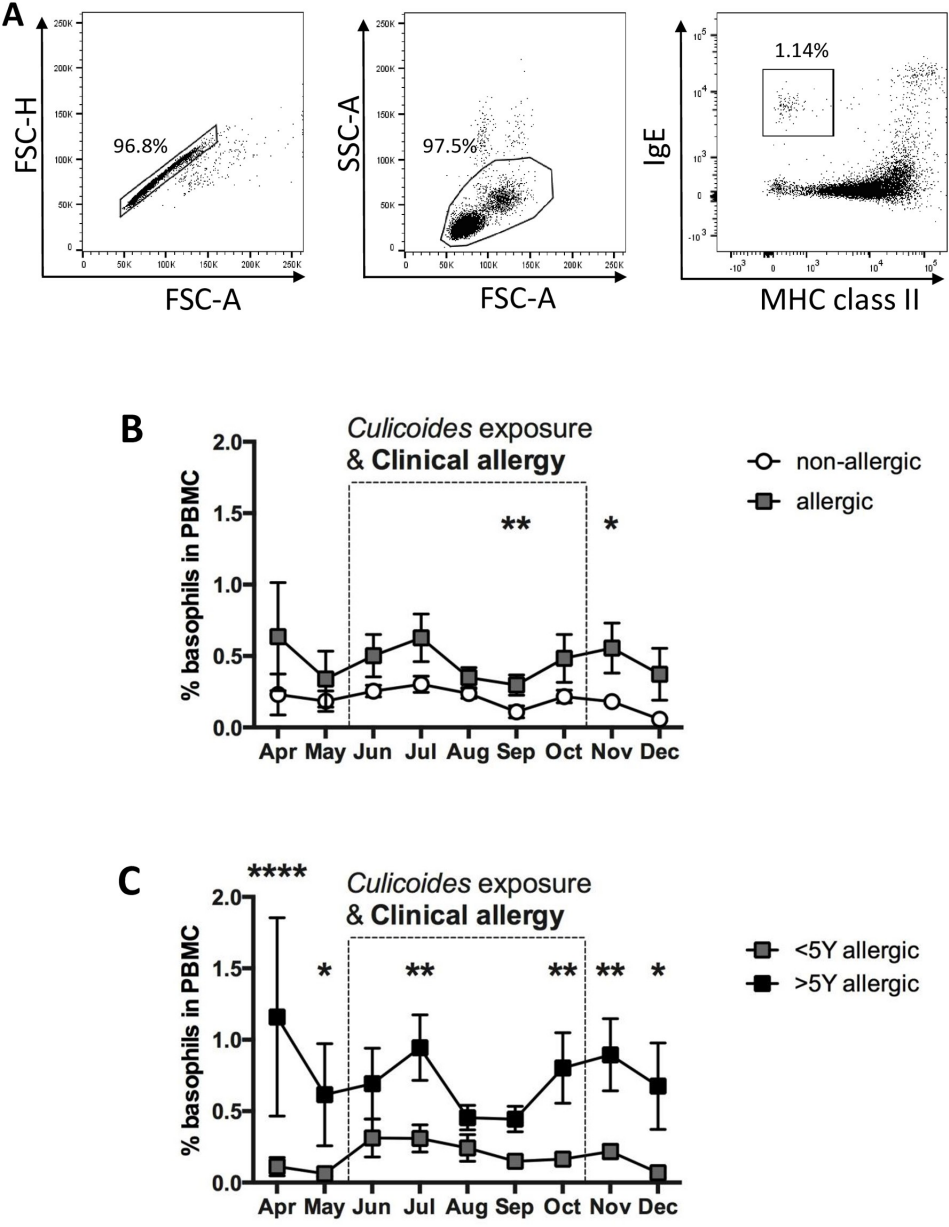
Basophils in the peripheral blood of allergic and non-allergic horses were compared by flow cytometric analysis. PBMC from sixteen Icelandic horses were analyzed once a month with cell surface staining for IgE and MHC class II. A) For analysis of basophils, cells were gated for doublet exclusion (left) and by forward (FSC) and side scatter (SSC) characteristics (middle). MHCII expression and IgE on gated PBMC was used to gate on basophils (IgE^+^/MHCII^low^ cells; right). B) Percentages of basophils were compared over time between allergic (n = 8) and non-allergic (n = 8) horses and C) in horses with allergy for >5 years (n = 4) and those with allergy <5 years (n = 4). The dotted box marks the period of exposure to *Cul* and clinical signs in the allergic horses. Data represent means and standard errors. Horse groups are compared by a non-parametric Mann Whitney Test. * *p*<0.05; ** *p*<0.01 **** *p* < 0.0001.

Peripheral blood cells producing IL-4 in response to stimulation with anti-IgE were previously characterized as IgE^+^/MHCII^low^ basophils [48, 50]. To explore if basophil numbers changed during the time of this study, we evaluated basophil percentages in PBMC by cell surface staining and flow cytometric analysis (Fig 3A). IgE^+^/MHCII^low^ basophils were present in low percentages in all horses at all times. Allergic horses had a trend towards higher basophil percentages throughout the study (Fig 3B). Significantly more basophils were found in the allergic group while horses showed clinical signs of chronic allergy in September (*p* = 0.0057), and when clinical signs started to resolve in November (*p* = 0.0443). In addition, horses that were allergic for more than five years (n = 4) showed overall higher basophil percentages in their peripheral blood than those that were allergic for less than five years (n = 4; Fig 3C). In fact, basophil percentages in horses with signs of allergy for less than five years closely resembled the cell numbers in non-allergic horses (Fig 3B and 3C). Significantly more basophils were found in horses with long-term CH in April (*p* < 0.0001), May (*p* = 0.0258), July (*p* = 0.0092), October (*p* = 0.0089), November (*p* = 0.0052) and December (*p* = 0.00134) (Fig 3C). IL-4 secretion data from PBMC of these horses were subsequently analyzed by separating IL-4 concentrations from horses with long-term (>5 years) and short-term allergy (<5 years). Overall, horses suffering from allergy for more than 5 years trended towards higher IL-4 production after stimulation of their PBMC with anti-IgE or *Cul* allergen (S2 Fig), while IL-4 secretion from PBMC of horses with short-term allergy resembled values of non-allergic horses (Fig 1). Our results suggest that peripheral blood basophil percentages are generally similar between allergic and non-allergic horses, increases in basophil percentages occur after longer duration of CH, and these basophils may contribute to IL-4 production after IgE crosslinking by *Cul* allergen. To test the latter conclusion, we next phenotyped the IL-4^+^ PBMC after *Cul* stimulation.

### Phenotyping of IL-4^+^ cells in response to *Cul* stimulation

**Fig 4:**
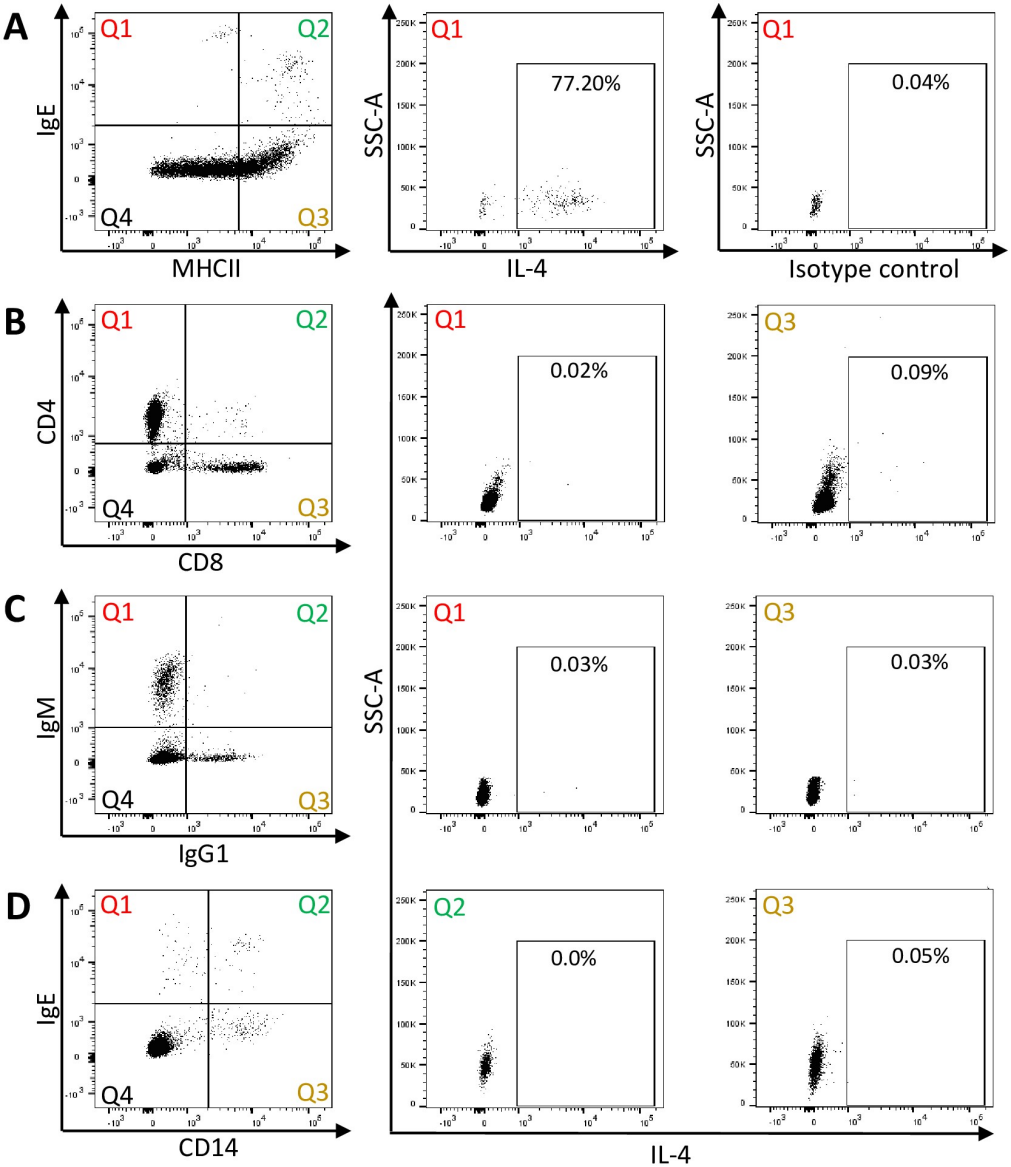
Phenotyping of IL-4^+^ peripheral blood cells after stimulation with *Cul* extract *in vitro*. PBMC were stimulated with *Cul* in presence of secretion blocker Brefeldin A for 4 hours. For flow cytometry analysis, cells were fixed and stained for intracellular IL-4 and subsequently for extracellular surface proteins: A) IgE and MHCII expression of PBMC to identify basophils in Quadrant (Q)1 (left), IL-4 production by IgE^+^/MHCII^low^ basophils (middle) and an intracellular isotype control staining (right); B) CD4 and CD8 expression to identify T cells (left), IL-4 production by CD4^+^ T cells in Q1 (middle), and by CD8^+^ T cells in Q3 (right); C) IgM and IgG expression to identify B cells (left), IL-4 production by IgM^+^ B cells (middle), and by IgG1^+^ cells (right); D) IgE and CD14 expression to identify monocytes (left), IL-4 production by IgE^+^/CD14^+^ IgE-binding monocytes (middle), and by IgE^-^/CD14^+^ monocytes (right). The IL-4^+^ cells give the percentages from the respective quadrant analysis. Representative flow cytometry plots from one horse in August are shown. The analysis was performed on all 16 horses.

In order to investigate if peripheral blood basophils and/or T-cells produce IL-4 in response to *Cul* stimulation, PBMC samples from all horses were stimulated for four hours *in vitro*, fixed and stained for intracellular IL-4 and cell surface markers. The cells were analyzed by flow cytometry. Phenotyping of PBMC showed that the majority of basophils produced IL-4 after *Cul* stimulation (Fig 4A). In contrast, only small percentages of IL-4^+^/CD4^+^ T-cells were found in response to *Cul* stimulation (Fig 4B, middle). Similarly, IL-4 production was not induced in CD8^+^ T-cells (Fig 4B, right), IgM^+^ or IgG1^+^ B-cells (Fig 4C), or CD14^+^ monocytes, independent whether the monocytes were surface IgE^+^ or not (Fig 4D).

The IL-4^+^ cells described in the initial time kinetics studies above (Fig 2B and 2C) were also further analyzed after staining of the cells with cell surface markers for basophils, T-cells and B-cells (S3 Fig). During the first 48 hours of *Cul* stimulation with Brefeldin A, IL-4 production was not observed in T-cells or B-cells (S3A and S3B Fig). IL-4^+^ basophils were observed as early as two hours post stimulation, increased until eight hours, stayed at a constant percentage until 16 hours, and declined afterwards (S3C Fig). When Brefeldin A was only added for the last four hours of incubation, highest IL-4^+^ basophil percentages were found at four hours of *Cul* stimulation (S3D Fig) similar to the IL-4^+^ cells described in Fig 2C.

### IL-4 production in response to short-term *Cul* stimulation is elevated in basophils from allergic horses but not in CD4^+^ T-cells

**Fig 5:**
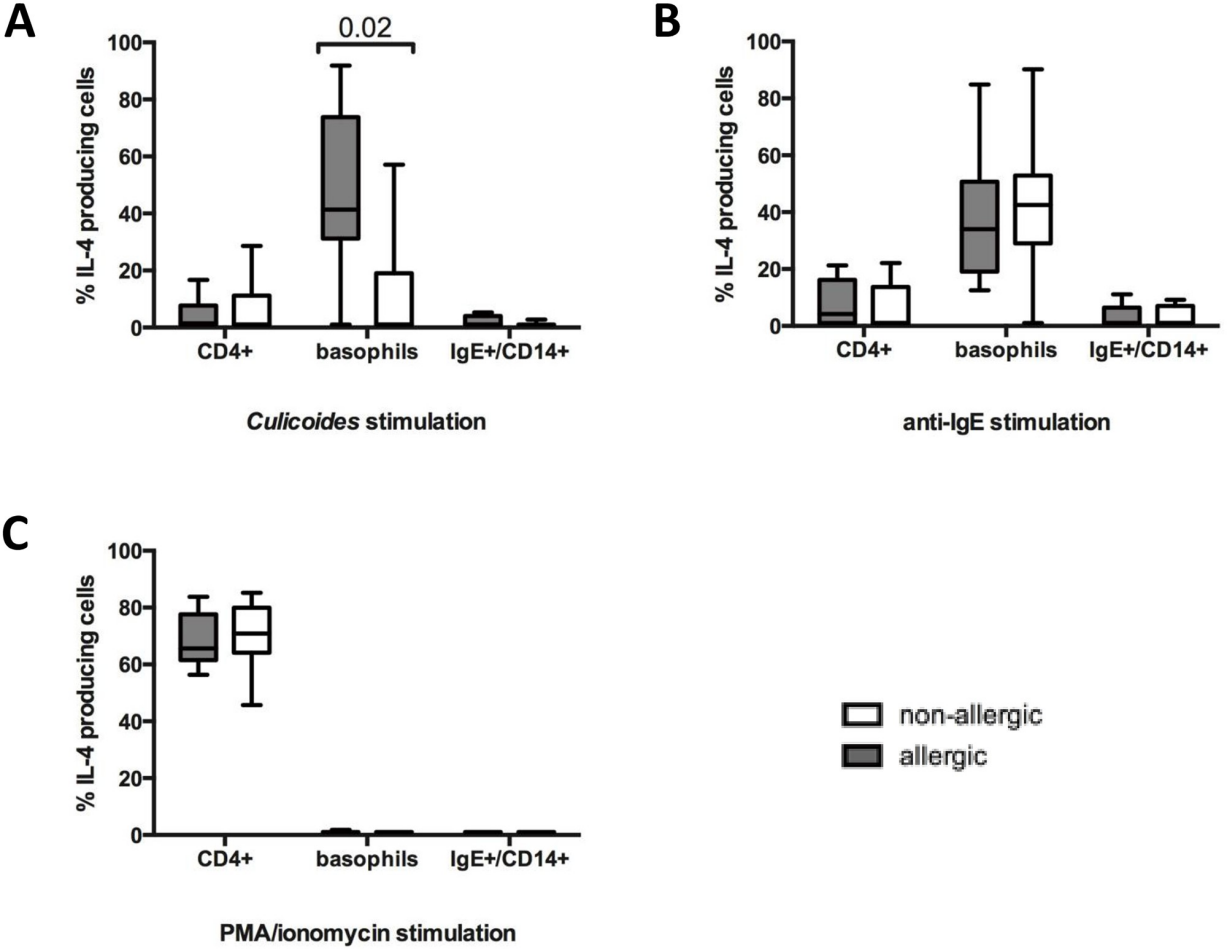
IL-4 production in CD4^+^ T-cells, basophils, and CD14^+^/IgE^+^ monocytes in horses with CH and non-allergic horses. PBMC from allergic (n = 8) and non-allergic horses (n = 8) were isolated in August and stimulated *in vitro* in presence of secretion blocker Brefeldin A for 4 hours with either A) *Cul* extract, B) anti-IgE-134, or C) PMA/ionomycin. Cells were stained for intracellular IL-4 production and cell surface markers and analyzed by flow cytometry analysis.

IL-4 production of different cell types in PBMC was compared after *Cul* stimulation for four hours between the allergic and non-allergic groups. Significantly higher percentages of IL-4^+^ basophils were found in allergic horses than in non-allergic horses (p = 0.0200; Fig 5A). IL-4 production in CD4^+^ T-cells after *Cul* stimulation was overall lower than in basophils and similar between horses with and without CH. IgE^+^ monocytes produced only minor or no IL-4 in both groups.

Stimulation of PBMC with anti-IgE again induced similar percentages of IL-4^+^ cells in both groups of horses and the majority of IL-4 production in all horses originated from basophils (Fig 5B). In contrast, IL-4^+^/CD4^+^ T-cells were the primary source of IL-4 after PMA/ionomycin stimulation (Fig 5C) confirming that the T-cells of all horses were fully capable of producing IL-4. Individual horse IL-4^+^ basophils and IL-4^+^/CD4^+^ T-cell percentages are shown for the different stimuli in S4 and S5 Tables, respectively.

All horses, regardless of CH diagnosis, did not produce IL-4 in their CD8^+^ T-cells, IgE^-^/CD14^+^ monocytes, and IgM^+^ B-cells after stimulation with *Cul*, anti-IgE, or PMA/ionomycin (S4 Fig).

## Discussion

IL-4 is a major modulator in the development of IgE-mediated allergies [22, 23]. It plays a crucial role in polarizing the immune response towards Th2 cell maturation and class switching of B-cells to produce IgE [23, 24, 26], which mediates mast cell and basophil sensitization [61–63]. In this article, we analyzed IL-4 production from PBMC of allergic horses with CH, an IgE-mediated seasonal allergic dermatitis that is induced by allergens from *Cul* midges. IL-4 secretion and IL-4^+^ peripheral blood cells from horses with CH and clinically healthy horses were analyzed during a period of nine months. During this longitudinal approach all horses lived in the same environment. For about five months, all horses were naturally exposed to *Cul*. For the remaining four months, the midges were not present in the environment and all horses appeared clinically healthy. This provided us with a unique model to study IL-4 in a controlled group of horses with and without exposure to *Cul* allergens. It also distinguishes our current approach from previous work using study populations of horses from several locations [64, 65]. The latter leads to multiple confounders and variations in respect to housing conditions, *Cul* exposure, medical and allergy treatment history, anthelminthic treatments, diets, sampling times etc. which can all influence immune responses including those related to CH.

Here, we reported that allergic horses have an overall trend towards higher IL-4 production in their PBMC after stimulation with *Cul* extract in comparison to non-allergic horses. We also observed significantly increased *Cul*-induced IL-4 production in allergic horses during the months when *Cul* midges were not present in the environment. This is unlike previous findings, where a difference was observed only during allergen exposure, and not when *Cul* midges were absent from the environment [64, 65]. In the previous study, horses were sampled only once during allergen exposure, and once in the winter time. *Cul* allergen exposure lasts usually five to six months and our findings here showed that *Cul*-specific IL-4 responses vary during this time period. However, IL-4 production by allergic and non-allergic horses was comparable in our controlled study while *Cul* midges were present in the environment. We hypothesize that during environmental exposure to *Cul* midges, immune cells quickly become exhausted at the local sites of inflammation in the skin of allergic horses due to continuous allergen stimulation. The same effect of exhaustion caused by continuous allergen-specific stimulation may also lead to a lack of differences in IL-4 production in peripheral blood cells from allergic and non-allergic horses during environmental *Cul* exposure. When the midges disappear from the environment, the cells can recover and/or are not triggered anymore by the constant environmental allergen challenge, and the difference in IL-4 production capacities between allergic and non-allergic horses becomes detectable again in PBMC.

When we stimulated PBMC with a crosslinking anti-IgE mAb, IL-4 production was similar in allergic and non-allergic horses. Besides circulating IgE specific for allergens, horses also have IgE antibodies against various parasites, e.g. gut nematodes in their serum [66, 67]. Thus, IgE-receptor expressing cells, such as basophils, bind IgE with specificities to a variety of antigens to their surfaces [67–70]. Allergen-specific IgE represents only a subfraction of the receptor bound IgE on basophils and crosslinking with anti-IgE resulted in similar secretion of IL-4 from cells of both groups independent on their allergy status. This suggested that PBMC from non-allergic horses and those with CH are equally capable of producing IL-4. In contrast, stimulation with *Cul* only crosslinked IgE antibodies specific for *Cul* on basophil surfaces, resulting in the seasonal differences in allergic and non-allergic horses discussed above.

Previous studies reported that peripheral T-cells from horses with CH produce higher amounts of IL-4 compared to non-allergic horses after *Cul* stimulation [64, 65]. Here, we observed that elevated concentrations of IL-4 are secreted from peripheral blood cells of allergic horses and that these cells are mainly IgE^+^/MHCII^low^ basophils. Increased percentages of IL-4^+^ T-cells were not observed in our experiments after *Cul* stimulation for up to 48 hours, while PMA/ionomycin stimulation confirmed that T-cells from the same horses expressed high amounts of IL-4. The seemingly discrepant results of our and previous studies can have several reasons. First, different horses and *Cul* preparations were used for stimulation which could have resulted in different outcomes. Second, PBMC in former studies were incubated for four days prior to the flow cytometric analysis, while our *Cul* stimulation was performed for four and in some instances for up to 48 hours. It may also be noteworthy that, in our experience, cell viability suffered from longer incubation with Brefeldin A. The cell morphology and viability changes after more than 12 hours of incubation with the secretion blocker made the cellular analysis of long-term PBMC cultures challenging. Our approach thus targeted the response of innate IgE-receptor bearing immune cells in the peripheral blood, such as basophils, while the former studies attempted to re-stimulate *Cul*-specific peripheral T-cells. Although the early IL-4 in response to *Cul* allergen is originating from basophils and T-cell responses could not be detected in our study during the first 48 hours of *Cul* stimulation, it is still possible that IL-4 production by T-cells starts after longer stimulation and as reported previously [64, 65]. Our finding that basophils produce early IL-4, shortly after allergen stimulation, is also consistent with work in murine allergy models, which show basophils as the primary source of the initial IL-4 during allergic responses [43, 44, 47, 71].

Rodent models have highlighted that basophils play a critical role in allergic inflammation and that this response is IgE dependent [61]. Studies in mice and human patients also highlighted that basophils can infiltrate to the site of inflammation and release inflammatory mediators including cytokines, chemokines, and proteases [61, 72–74]. In addition, basophils can play an IgE independent role in allergies by producing IL-4 in response to other stimuli than the specific allergens [61, 75]. The role of basophils as a primary source of initial IL-4 secretion that further stimulates IL-4 production by other immune cells, e.g. Th2-cells, type 2 innate lymphoid cells, and eosinophils has been discussed previously. Basic studies in mice supported that basophils are essential innate immune regulatory cells during allergic inflammation and basophil derived IL-4 provides the initial signal leading to Th2-cell polarization *in vivo* [43, 44, 47, 71]. Allergy studies in humans have also shown increased basophil infiltration at site of inflammation, including the skin and mucosal surfaces, in allergic individuals [76–78]. Here, we showed that horses with CH overall trended towards increased percentages of basophils in peripheral blood compared to healthy controls, which is consistent with human and murine allergy studies [76–78]. Our data also suggest that horses that were affected with CH for five years or longer had significantly higher basophil percentages in their peripheral blood than horses with a shorter history of CH (<5 years). It is currently unknown whether basophils in horses allergic for more than 5 years are surviving longer, or if repeated clinical allergy induces enhanced basophil production in long-term CH affected horses. Previous studies have shown that IgE can induce increased basophil numbers in murine models [49, 78]. Additional future studies will further enhance our understanding of the role of basophils in horses with CH.

## Conclusions

In summary, our current data indicate that peripheral blood basophils of CH allergic horses secrete increased amounts of IL-4 following *Cul* specific stimulation *in vitro* when *Cul* midges are not in the horses’ environment. During the summer, IL-4 production is similar in all horses, suggesting a stage of exhaustion from chronic inflammation in allergic horses. In horses with clinical allergy for five or more years, higher percentages of basophils circulate in peripheral blood compared to healthy horses. We also report that basophils are the major innate source of IL-4 after allergen-specific stimulation of PBMC from horses with CH, and basophils from healthy horses produce significantly less IL-4 in response to *Cul* allergen. This is the first report on a potential role for basophils in the development and maintenance of allergic inflammation during CH. Basophils may serve as a new cellular target for designing novel strategies to prevent or treat CH in horses.

## Supporting information

S1 Fig IL-4 secretion form PBMC of allergic and non-allergic horses after 48 hours of stimulation with anti-IgE or *Cul* extract *in vitro*. Blood samples were obtained from allergic (n = 8) and non-allergic horses (n = 8) once a month between April and December. The dotted box indicates the period of environmental exposure to *Cul* when horses with CH also showed clinical signs of allergy. PBMC were stimulated *in vitro* with different stimuli, cell culture supernatants were harvested after 48 hours of incubation, and IL-4 was measured in the supernatants using s fluorescent bead-based IL-4 assay. IL-4 secretion from PBMC after stimulation with A) anti-IgE, clone 134, which crosslinks IgE on the cell surface, and B) *Cul* extract. Graphs represent means with standard errors. Allergic and non-allergic groups were compared using non-parametric Mann Whitney tests. Differences in IL-4 secretion between allergic and non-allergic groups were not observed after 48 hours of incubation.
(TIF)

S2 Fig IL-4 secretion from PBMC after stimulation with anti-IgE or *Cul* allergen in horses with long-term or short-term allergy. Blood samples were obtained monthly from allergic horses with clinical allergy for more than five previous years (>5 years; n = 4) and less than five years (<5 years; n = 4) from April to December. The period of environmental exposure to *Cul* and clinical signs of allergy is indicated by the dotted box. PBMC were stimulated *in vitro* with A) the crosslinking anti-IgE mAb 134, or B) *Cul* extract. Cell culture supernatants were harvested after 24 hours of incubation and IL-4 was measured using a fluorescent bead-based assay. Graphs represent means with standard errors. Allergic and non-allergic groups were compared using non-parametric Mann Whitney tests. Differences in IL-4 secretion between the two allergic groups were not significant after 24 hours of incubation.
(TIF)

S3 Fig IL-4^+^ cell phenotyping in equine PBMC after stimulation with *Cul* extract for 2 to 48 hours. PBMC from horses were isolated and stimulated with *Cul* extract *in vitro* in the presence of the secretion blocker Brefeldin A. After incubation, the cells were fixed, stained for intracellular IL-4 production together with different cell surface markers, and measured by flow cytometric analysis. The graphs represent percentages of A) IL-4^+^ B-cells, (B) IL-4^+^ T-cells and C) IL-4^+^ basophils. PBMC from allergic horses (n = 2) were harvested after 2, 4, 8, 16, 20, 24, 28, 32, 40, or 48 hours of incubation with Brefeldin A present in the culture for the entire stimulation time. D) PBMC from allergic (n = 5) and non-allergic horses (n = 5) were stimulated for different times between 4 and 48 hours with Brefeldin A only present during the last 4 hours of incubation. Graphs represent means with standard errors.
(TIF)

S4 Fig Flow cytometry analysis of IL-4 producing cells in IgM^+^ B-cell, CD8^+^ T-cell, and IgE^-^/CD14^+^ monocyte populations. PBMC from eight allergic Icelandic horses and eight non-allergic horses were stimulated with A) *Cul* extract, B) anti-IgE 134 and C) PMA/ionomycin in presence of secretion blocker Brefeldin A for 4 hours. Afterwards, the cells were fixed, stained for intracellular IL-4 and different cell surface marker, and were analyzed in a flow cytometer. The graphs represent means and standard errors of relative percentages of IL-4^+^ cells.
(TIF)

S1 Table Clinical allergy scoring system. (DOCX)

S2 Table Clinical allergy scores ^a^ of the horses between January to April, November and December when *Culicoides* was not present in the environment of the horses. (DOCX)

S3 Table IL-4 concentrations in PBMC supernatants from allergic and clinically healthy control horses after 24 and 48 hours of *Culicoides (Cul)* stimulation *in vitro*. (DOCX)

S4 Table Percentages of IL-4^+^ basophils out of total IL-4^+^ cells in PMBC of allergic horses with *Culicoides* hypersensitivity and clinically healthy control horses after *in vitro* stimulation. (DOCX)

S5 Table Percentages of IL-4^+^/CD4^+^ T-cells out of total IL-4^+^ cells in PMBC of allergic horses with *Culicoides* hypersensitivity and clinically healthy control horses after *in vitro* stimulation. (DOCX)
