# Supplementary figures and images for: Peripheral blood basophils are the main source for early interleukin-4 secretion upon in vitro stimulation with Culicoides allergen in allergic horses
Source: PLoS One. 2021 May 26;16(5):e0252243. doi: 10.1371/journal.pone.0252243 (PMC8153460; doi:10.1371/journal.pone.0252243)

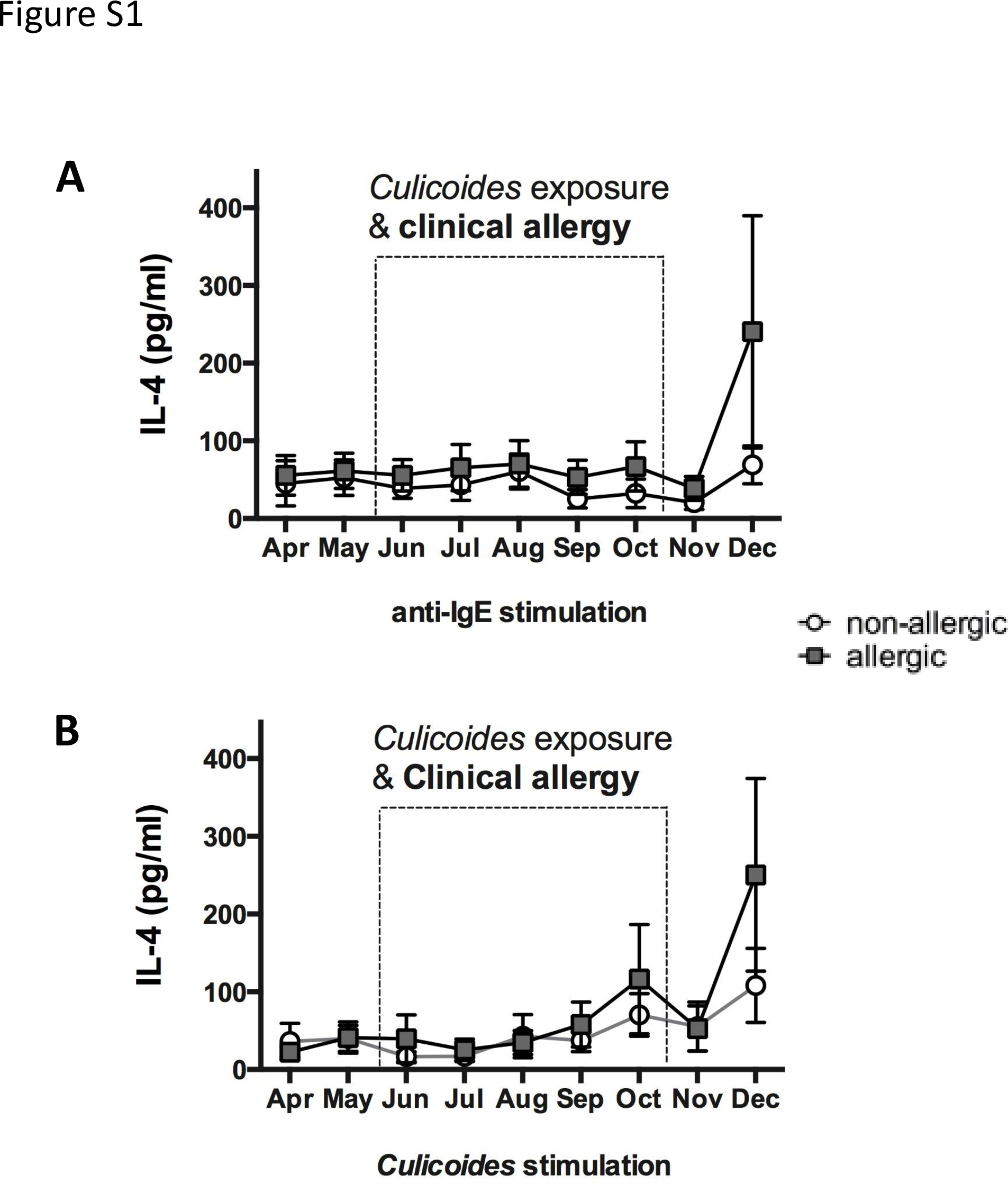

Supplement: S1 Fig — Blood samples were obtained from allergic (n = 8) and non-allergic horses (n = 8) once a month between April and December. The dotted box indicates the period of environmental exposure to Cul when horses with CH also showed clinical signs of allergy. PBMC were stimulated in vitro with different stimuli, cell culture supernatants were harvested after 48 hours of incubation, and IL-4 was measured in the supernatants using s fluorescent bead-based IL-4 assay. IL-4 secretion from PBMC after stimulation with A) anti-IgE, clone 134, which crosslinks IgE on the cell surface, and B) Cul extract. Graphs represent means with standard errors. Allergic and non-allergic groups were compared using non-parametric Mann Whitney tests. Differences in IL-4 secretion between allergic and non-allergic groups were not observed after 48 hours of incubation. (TIF) [file pone.0252243.s001.tif]

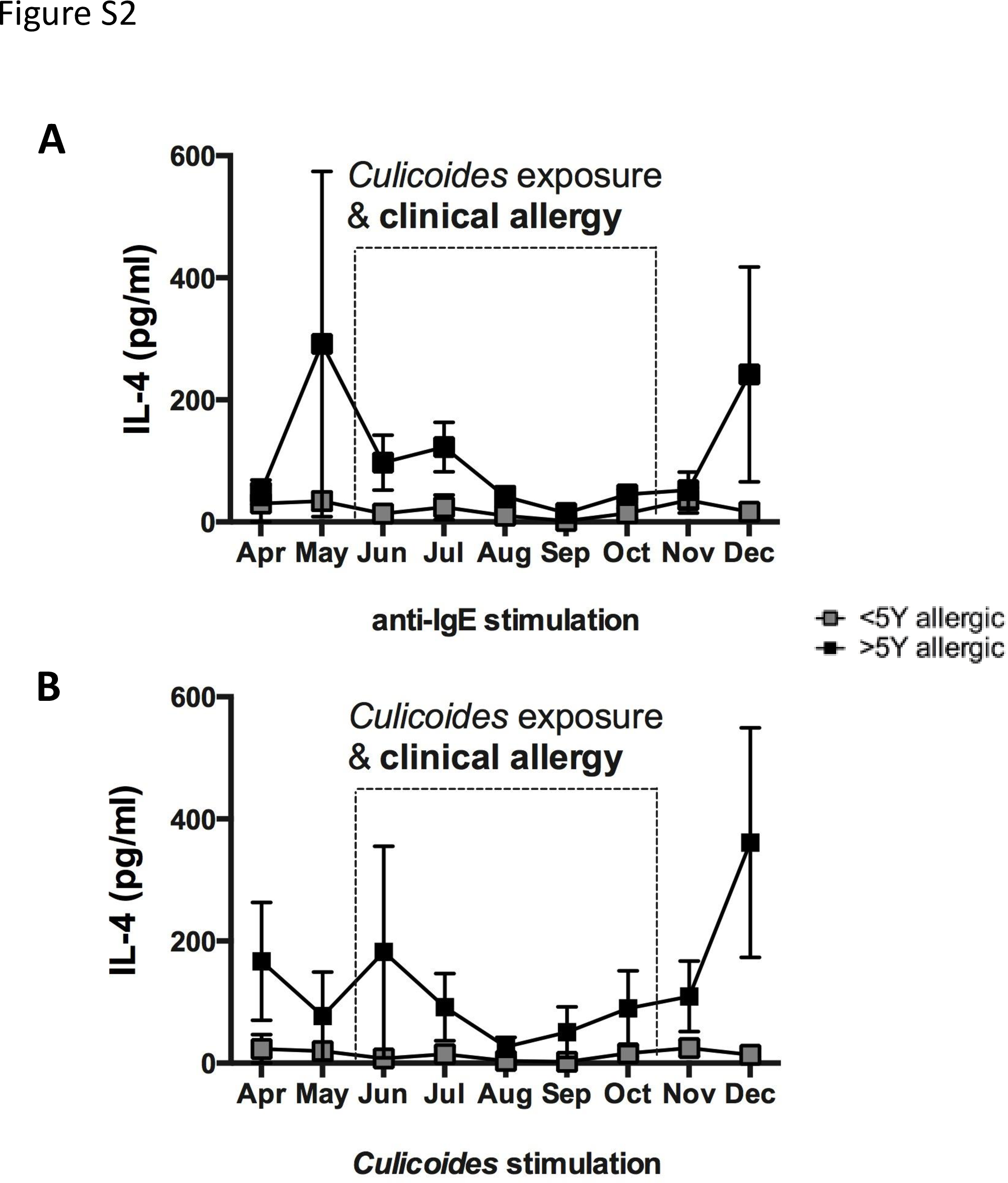

Supplement: S2 Fig — Blood samples were obtained monthly from allergic horses with clinical allergy for more than five previous years (>5 years; n = 4) and less than five years (<5 years; n = 4) from April to December. The period of environmental exposure to Cul and clinical signs of allergy is indicated by the dotted box. PBMC were stimulated in vitro with A) the crosslinking anti-IgE mAb 134, or B) Cul extract. Cell culture supernatants were harvested after 24 hours of incubation and IL-4 was measured using a fluorescent bead-based assay. Graphs represent means with standard errors. Allergic and non-allergic groups were compared using non-parametric Mann Whitney tests. Differences in IL-4 secretion between the two allergic groups were not significant after 24 hours of incubation. (TIF) [file pone.0252243.s002.tif]

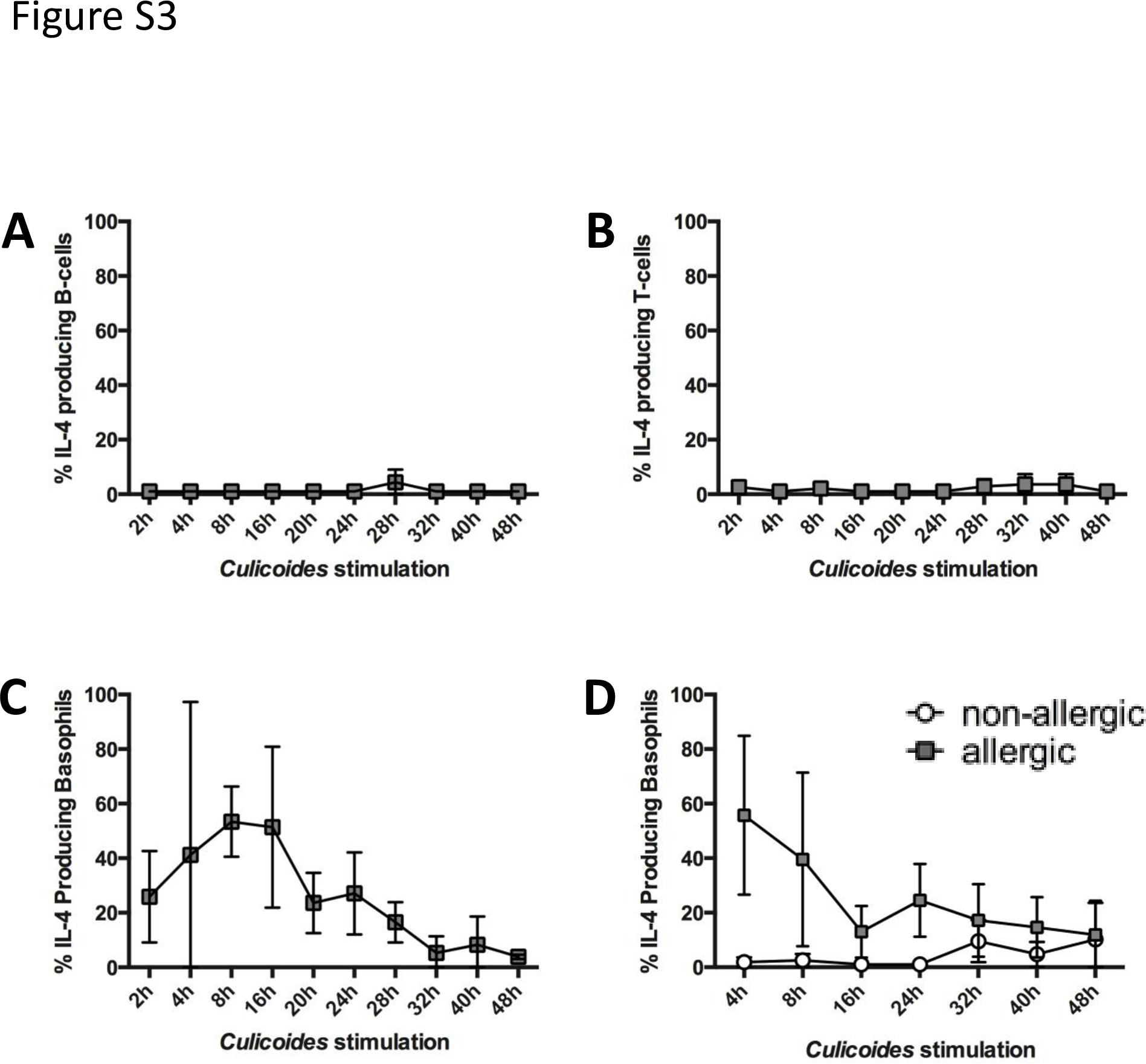

Supplement: S3 Fig — PBMC from horses were isolated and stimulated with Cul extract in vitro in the presence of the secretion blocker Brefeldin A. After incubation, the cells were fixed, stained for intracellular IL-4 production together with different cell surface markers, and measured by flow cytometric analysis. The graphs represent percentages of A) IL-4+ B-cells, (B) IL-4+ T-cells and C) IL-4+ basophils. PBMC from allergic horses (n = 2) were harvested after 2, 4, 8, 16, 20, 24, 28, 32, 40, or 48 hours of incubation with Brefeldin A present in the culture for the entire stimulation time. D) PBMC from allergic (n = 5) and non-allergic horses (n = 5) were stimulated for different times between 4 and 48 hours with Brefeldin A only present during the last 4 hours of incubation. Graphs represent means with standard errors. (TIF) [file pone.0252243.s003.tif]

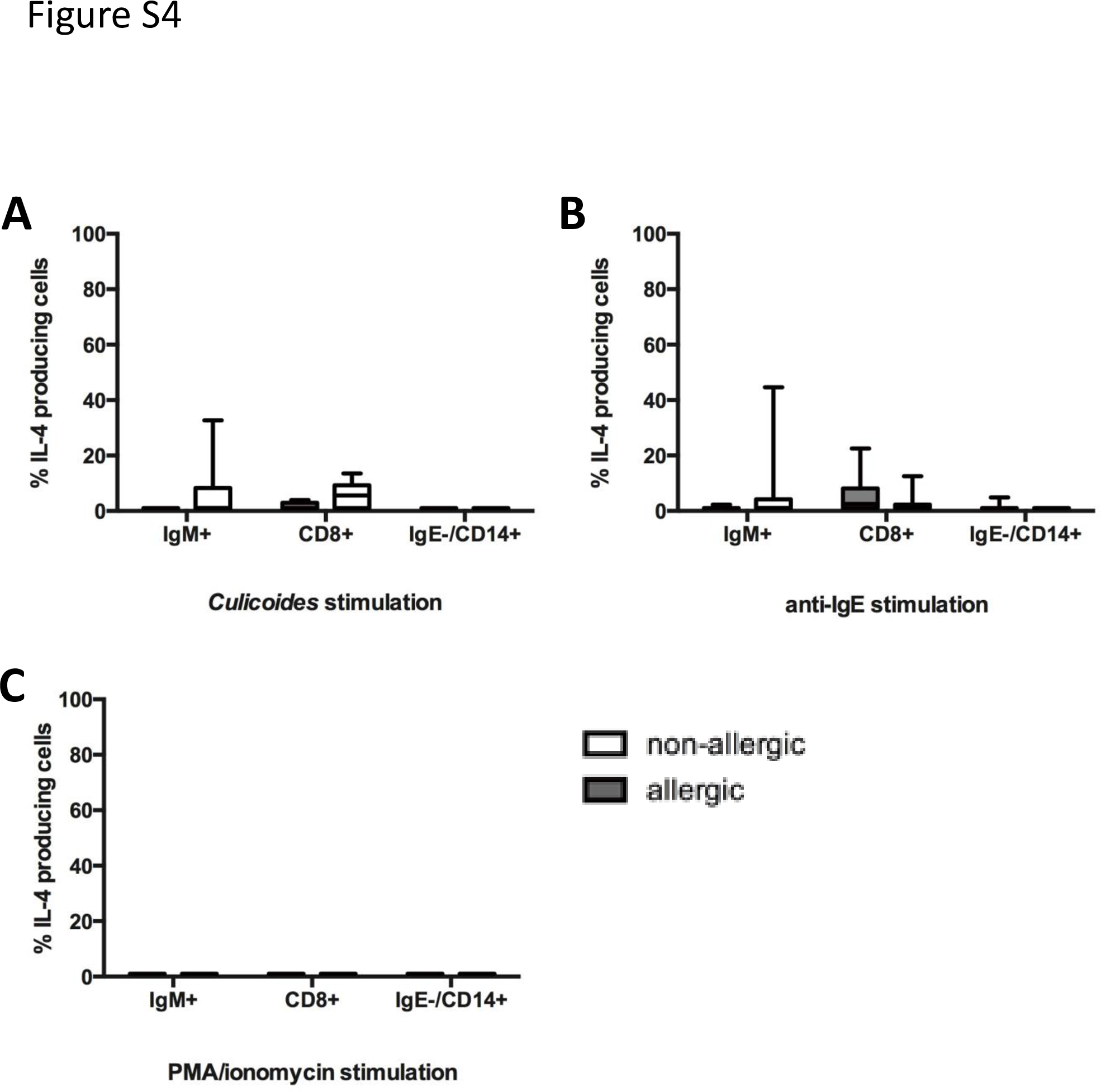

Supplement: S4 Fig — PBMC from eight allergic Icelandic horses and eight non-allergic horses were stimulated with A) Cul extract, B) anti-IgE 134 and C) PMA/ionomycin in presence of secretion blocker Brefeldin A for 4 hours. Afterwards, the cells were fixed, stained for intracellular IL-4 and different cell surface marker, and were analyzed in a flow cytometer. The graphs represent means and standard errors of relative percentages of IL-4+ cells. (TIF) [file pone.0252243.s004.tif]
