# Supplementary material for: Peripheral blood basophils are the main source for early interleukin-4 secretion upon in vitro stimulation with Culicoides allergen in allergic horses
Source: PLoS One. 2021 May 26;16(5):e0252243. doi: 10.1371/journal.pone.0252243 (PMC8153460; doi:10.1371/journal.pone.0252243)
Supplement: S1 Table — (DOCX) [file pone.0252243.s005.docx]

**S1 Table. Clinical allergy scoring system.**

| **Clinical sign** | **Grade** | **Score** | **Maximal score** |
| --- | --- | --- | --- |
| **Pruritus** | No mane or tail scratching | 0 | 3 |
|  | Mild mane and/or tail scratching | 1 |  |
|  | Moderate mane and/or tail scratching | 2 |  |
|  | Intense mane and tail scratching | 3 |  |
| **Alopecia** | None | 0 | 4 |
|  | Few broken hairs one location | 1 |  |
|  | Several locations with broken hairs | 2 |  |
|  | Moderate hair loss, mane or tail | 3 |  |
|  | Severe hair loss, mane and tail | 4 |  |
| **Skin irritation** | No skin irritation | 0 | 3 |
|  | Mild dermatitis, one location | 1 |  |
|  | Moderate dermatitis, several locations | 2 |  |
|  | Dermatitis with skin lesions | 3 |  |
|  | **Total score** |  | 10 |

The clinical allergy scoring system is described in more detail by Miller et al. 2019 [51].
