# Supplementary material for: Peripheral blood basophils are the main source for early interleukin-4 secretion upon in vitro stimulation with Culicoides allergen in allergic horses
Source: PLoS One. 2021 May 26;16(5):e0252243. doi: 10.1371/journal.pone.0252243 (PMC8153460; doi:10.1371/journal.pone.0252243)
Supplement: S2 Table — (DOCX) [file pone.0252243.s006.docx]

**S2 Table. Clinical allergy scores ^a^ of the horses between January to April, November and December when *Culicoides* was not present in the environment of the horses.**

|  | **Horses** | **Sex** | **Year of birth** | **Age ^b^** | **Allergy score ^c^ without *Culicoides* exposure** | | | | | | **Median (range)** |
| --- | --- | --- | --- | --- | --- | --- | --- | --- | --- | --- | --- |
|  |  |  |  |  | **JAN** | **FEB** | **MAR** | **APR** | **NOV** | **DEC** |  |
| **Allergic** | 1 | Mare | 2005 | 13 | 0.0 | 0.0 | 0.0 | 0.0 | 0.0 | 0.0 | 0.0 (0) |
|  | 2 | Mare | 2004 | 14 | 0.7 | 0.0 | 0.0 | 0.0 | 1.0 | 1.0 | 0.3 (0-1) |
|  | 3 | Mare | 2004 | 14 | 0.7 | 0.0 | 0.0 | 0.0 | 1.0 | 0.0 | 0.0(0-1) |
|  | 4 | Mare | 2003 | 15 | 1.0 | 0.0 | 0.0 | 0.0 | 1.0 | 1.0 | 0.5(0-1) |
|  | 5 | Mare | 2005 | 13 | 0.3 | 0.0 | 0.0 | 0.0 | 1.0 | 0.0 | 0.0 (0-1) |
|  | 6 | Mare | 2009 | 9 | 0.0 | 0.0 | 0.0 | 0.0 | 1.0 | 0.0 | 0.0 (0-1) |
|  | 7 | Mare | 2011 | 7 | 0.0 | 0.0 | 0.0 | 0.0 | 0.0 | 0.0 | 0.0 (0) |
|  | 8 | Gelding | 2011 | 7 | 0.0 | 0.0 | 0.0 | 0.0 | 0.0 | 0.0 | 0.0 (0) |
| **Non-allergic** | 9 | Mare | 2007 | 11 | 0.0 | 0.0 | 0.0 | 0.0 | 0.0 | 0.0 | 0.0 (0) |
|  | 10 | Mare | 2006 | 12 | 0.0 | 0.0 | 0.0 | 0.0 | 0.0 | 0.0 | 0.0 (0) |
|  | 11 | Mare | 2013 | 5 | 0.0 | 0.0 | 0.0 | 0.0 | 0.0 | 0.0 | 0.0 (0) |
|  | 12 | Mare | 2011 | 7 | 0.0 | 0.0 | 0.0 | 0.0 | 0.0 | 0.0 | 0.0 (0) |
|  | 13 | Mare | 2011 | 7 | 0.0 | 0.0 | 0.0 | 0.0 | 0.0 | 0.0 | 0.0 (0) |
|  | 14 | Mare | 2012 | 6 | 0.0 | 0.0 | 0.0 | 0.0 | 0.0 | 0.0 | 0.0 (0) |
|  | 15 | Mare | 2013 | 5 | 0.0 | 0.0 | 0.0 | 0.0 | 0.0 | 0.0 | 0.0 (0) |
|  | 16 | Mare | 2013 | 5 | 0.0 | 0.0 | 0.0 | 0.0 | 0.0 | 0.0 | 0.0 (0) |

^a^ Clinical allergy scores ranged from 0-10 as described by Miller et al. 2019 [51].

^b^ Age in years at time of study, in 2018.

^c^ Average allergy score at time of study, in 2018.
