# Supplementary material for: Peripheral blood basophils are the main source for early interleukin-4 secretion upon in vitro stimulation with Culicoides allergen in allergic horses
Source: PLoS One. 2021 May 26;16(5):e0252243. doi: 10.1371/journal.pone.0252243 (PMC8153460; doi:10.1371/journal.pone.0252243)
Supplement: S3 Table — (DOCX) [file pone.0252243.s007.docx]

**S3 Table. IL-4 concentrations in PBMC supernatants from allergic and clinically healthy control horses after 24 and 48 hours of *Culicoides (Cul)* stimulation *in vitro*.**

| **IL-4 concentration, median (range)** | | | | |
| --- | --- | --- | --- | --- |
|  | ***Cul* stimulation for 24 hours** | | ***Cul* stimulation for 48 hours** | |
| **Months** | **Allergic (n=8)** | **Non-allergic (n=8)** | **Allergic (n=8)** | **Non-allergic (n=8)** |
| **April** | 47.3 (0-450) | 0.0 (0-62) | 25.2 (0-43) | 12.7 (0-196) |
| **May** | 7.8 (3-294) | 1.5 (0-36) | 12.8 (0-142) | 32.5 (2-150) |
| **June** | 10.0 (0-702) | 8.7 (1-46) | 12.2 (0-254) | 11.5 (0-55) |
| **July** | 30.5 (0-256) | 16.1 (0-30) | 8.7 (0-111) | 13.8 (0-55) |
| **August** | 0.0 (0-55) | 6.3 (0-26) | 15.5 (0-119) | 16.1 (0-233) |
| **September** | 4.3 (0-174) | 2.4 (0-50) | 19.4 (0-242) | 20.6 (0-121) |
| **October** | 25.5 (0-271) | 9.3 (3.7-83) | 36.8 (5-596) | 52.4 (5-240) |
| **November** | 31.3 (3.65-275) | 8.1 (0.9-20) | 27.8 (7-253) | 28.7 (0-271) |
| **December** | 35.7 (0-752) | 6.1 (0-29) | 77.7 (37-914) | 72.8 (24-434) |
