# Supplementary material for: Peripheral blood basophils are the main source for early interleukin-4 secretion upon in vitro stimulation with Culicoides allergen in allergic horses
Source: PLoS One. 2021 May 26;16(5):e0252243. doi: 10.1371/journal.pone.0252243 (PMC8153460; doi:10.1371/journal.pone.0252243)
Supplement: S4 Table — (DOCX) [file pone.0252243.s008.docx]

**S4 Table. Percentages of IL-4^+^ basophils out of total IL-4^+^ cells in PBMC of allergic horses with *Culicoides* hypersensitivity and clinically healthy control horses after *in vitro* stimulation.**

|  | **IL-4^+^ basophils (%) ^a^** | | | | | |
| --- | --- | --- | --- | --- | --- | --- |
| **Stimulation** | ***Culicoides* extract** | | **Anti-IgE 134** | | **PMA/ionomycin** | |
|  | **allergic** | **non-allergic** | **allergic** | **non-allergic** | **allergic** | **non-allergic** |
|  | 91.9 | 1.0 | 84.9 | 1.0 | 1.8 | 1.0 |
|  | 82.6 | 57.1 | 56.0 | 53.8 | 1.0 | 1.0 |
|  | 35.5 | 25.0 | 34.9 | 90.2 | 1.0 | 1.0 |
|  | 38.9 | 1.3 | 34.7 | 35.8 | 1.0 | 1.0 |
|  | 29.8 | 1.0 | 15.8 | 50.0 | 1.0 | 1.0 |
|  | 47.4 | 1.0 | 33.3 | 38.5 | 1.0 | 1.0 |
|  | 43.8 | 1.0 | 29.2 | 46.5 | 1.0 | 1.0 |
|  | 1.0 | 1.0 | 12.5 | 26.8 | 1.0 | 1.0 |
| **Median** | 41.4 | 1.0 | 34.0 | 42.5 | 1.0 | 1.0 |
| **Range** | 1.0-91.9 | 1.0-57.1 | 12.5-84.9 | 1.0-90.2 | 1.0-1.8 | 1.0-1.0 |

^a^ if the percentage of IL-4^+^ basophils out of total IL-4+ PBMC was equal to the isotype control a value of 1.0 was assigned
