# Supplementary material for: Peripheral blood basophils are the main source for early interleukin-4 secretion upon in vitro stimulation with Culicoides allergen in allergic horses
Source: PLoS One. 2021 May 26;16(5):e0252243. doi: 10.1371/journal.pone.0252243 (PMC8153460; doi:10.1371/journal.pone.0252243)
Supplement: S5 Table — (DOCX) [file pone.0252243.s009.docx]

**S5 Table: Percentages of IL-4^+^/CD4^+^ T-cells out of total IL-4^+^ cells in PBMC of allergic horses with *Culicoides* hypersensitivity and clinically healthy control horses after *in vitro* stimulation.**

|  | IL-4^+^/CD4^+^ T-cells (%) | | | | | |
| --- | --- | --- | --- | --- | --- | --- |
| Stimulation | *Culicoides* extract | | Anti-IgE 134 | | PMA/ionomycin | |
|  | allergic | non-allergic | allergic | non-allergic | allergic | non-allergic |
|  | 1.0 | 14.6 | 1.0 | 22.1 | 56.4 | 85.2 |
|  | 1.9 | 1.0 | 14.9 | 1.0 | 83.8 | 45.7 |
|  | 9.5 | 1.0 | 5.7 | 1.0 | 63.4 | 64.0 |
|  | 1.0 | 1.0 | 1.2 | 1.0 | 61.3 | 74.4 |
|  | 1.0 | 28.6 | 2.7 | 1.5 | 74.5 | 76.1 |
|  | 2.4 | 1.1 | 21.3 | 17.8 | 62.1 | 81.2 |
|  | 1.0 | 1.0 | 1.0 | 1.0 | 67.8 | 67.4 |
|  | 16.7 | 1.0 | 16.7 | 1.0 | 78.7 | 64.6 |
| Median | 1.5 | 1.0 | 4.2 | 1.0 | 65.6 | 70.9 |
| Range | 1.0-16.7 | 1.0-28.6 | 1.0-21.3 | 1.0-22.1 | 56.4-83.8 | 45.7-85.2 |

^a^ if the percentage of IL-4^+^/CD4+ T-cells out of total IL-4+ PBMC was equal to the isotype control a value of 1.0 was assigned
